# Prevalence and Antimicrobial Resistance Profiles of Bloodstream Pathogens in Feni, Bangladesh

**DOI:** 10.1155/bmri/1430236

**Published:** 2026-02-10

**Authors:** Samim Mia, Md Zahirul Islam, Mst Rahima Khatun, Md Jahid Hasan Dewan, Md. Obydur Rahman, Mohammad Zakerin Abedin

**Affiliations:** ^1^ Department of Microbiology, Vital Research, Feni, Bangladesh; ^2^ College of Life Science and Technology, Huazhong University of Science and Technology, Wuhan, China, hust.edu.cn; ^3^ College of Life Sciences, Northwest A&F University, Yangling, China, nwsuaf.edu.cn; ^4^ Faculty of Arts, National University, Gazipur, Bangladesh, inu.ac.kr; ^5^ Department of Microbiology, Jagannath University, Dhaka, Bangladesh, jagannathuniversity.org; ^6^ Department of Microbiology, Khwaja Yunus Ali University, Sirajganj, Bangladesh, kyau.edu.bd

**Keywords:** amikacin, antimicrobial resistance, bangladesh healthcare, bloodstream infections, gram-negative bacteria

## Abstract

**Background:**

Bloodstream infections cause substantial morbidity and mortality, increasingly exacerbated by antimicrobial resistance. We investigated the prevalence and resistance profiles of bloodstream pathogens in Feni, Bangladesh.

**Methods:**

Between October 2024 and March 2025, we conducted a 6‐month cross‐sectional study at a diagnostic center in Feni, Bangladesh. Blood cultures from 498 patients with suspected bloodstream infections were analyzed, and bacterial isolates were identified and tested for antibiotic susceptibility using standard methods.

**Results:**

Among 498 blood samples, 49 (9.84%) yielded positive bacterial growth. Infections were most common in patients aged over 49 years (36.7%), with near‐equal distribution between males (51.0%) and females (49.0%). Gram‐negative bacteria predominated (73.5%), including *Salmonella* spp. (28.6%), *Escherichia coli* (20.4%), and *Salmonella Typhi* (14.3%), whereas *Staphylococcus aureus* (10.2%) was the leading gram‐positive isolate. Gram‐negative isolates exhibited high resistance to ampicillin, amoxyclav, and cefuroxime, whereas amikacin maintained > 70% sensitivity. Gram‐positive isolates were largely resistant to third‐generation cephalosporins but were very susceptible to gentamicin and ciprofloxacin.

**Conclusion:**

This study reveals the predominance of gram‐negative bloodstream infections and extensive *β*‐lactam resistance in Feni, Bangladesh. Amikacin demonstrated the highest efficacy, supporting its empirical consideration pending susceptibility results. These findings highlight the urgent need for local antibiogram development and strengthened regional resistance surveillance.

## 1. Introduction

Bloodstream infections (BSIs) are severe clinical conditions arising from the invasion of pathogenic microorganisms into the circulatory system, often leading to sepsis and multi‐organ failure [1]. A diverse range of bacteria and fungi causes these infections, entering the bloodstream through surgical wounds, indwelling devices, or the spread of localized infections [2, 3]. Gram‐negative bacteria, including *Escherichia coli*, *Klebsiella* spp., and *Pseudomonas aeruginosa*, together with gram‐positive bacteria such as *Staphylococcus aureus* and *Enterococcus* spp., are the principal pathogens implicated [4]. The composition of these pathogens varies across healthcare settings, patient populations, and geographic regions [5]. Understanding their prevalence and antimicrobial resistance (AMR) profiles is essential for guiding effective therapy and infection control.

AMR is a growing global health crisis, driven by the misuse and overuse of antimicrobial agents in medical, agricultural, and community settings [6, 7]. Sub‐therapeutic dosing, self‐medication, and poor adherence to treatment exert selective pressure on microbes, fostering resistant strains [8–10]. The widespread use of antibiotics in livestock and aquaculture further accelerates this process by promoting resistance genes within commensal bacteria, which can transfer to human pathogens through the food chain or environmental pathways [11, 12]. Inadequate infection control, poor sanitation, and global travel facilitate the rapid spread of these microorganisms [13]. The burden is particularly severe in low‐resource regions, including South Asia, where limited access to healthcare, diagnostics, and regulation exacerbates the challenge [14]. These realities underscore the urgent need for stronger global stewardship and coordinated surveillance.

Bangladesh, like many low‐ and middle‐income countries, faces persistent challenges in the surveillance of BSIs. Limited diagnostic capacity, absence of routine antibiogram reporting, and irrational antibiotic use continue to drive the rise of AMR [14, 15]. Increasing AMR among major BSI pathogens such as *E. coli*, *Klebsiella pneumoniae,* and *S. aureus* has been documented, complicating treatment and worsening clinical outcomes [16]. Despite growing concern, region‐specific data on the bacterial spectrum and resistance trends of BSIs remain scarce, particularly outside major urban centers. Diagnostic facilities in semiurban regions like Feni serve large catchment populations and play a crucial role in infectious disease detection and monitoring of AMR transmission. Addressing these gaps requires strengthened surveillance, improved laboratory infrastructure, and antibiotic stewardship strategies tailored to local epidemiological contexts.

To address these gaps, we conducted a cross‐sectional study at a major referral diagnostic center in Feni, Bangladesh, with three key objectives (i) to determine the prevalence of bloodstream pathogens, (ii) to characterize their AMR profiles, and (iii) to identify the most effective antibiotics with consistent in vitro activity against these isolates. The findings provide essential microbiological evidence to inform regional antibiotic policies, optimize empirical therapy, and support antimicrobial stewardship in resource‐limited healthcare settings.

## 2. Materials and Methods

### 2.1. Study Design and Sample Collection

This cross‐sectional study was conducted at the Vital Research Unit‐1 in Feni, a reputable medical diagnostic center that provides extensive laboratory analyses and various healthcare diagnostic services to the community. Blood samples were obtained from patients clinically suspected of BSIs from 1 October 2024, to 31 March 2025, utilizing a consecutive sampling method that encompassed all eligible blood culture submissions during this timeframe. In total, 498 samples formed the complete sampling frame; therefore, no separate sample size calculation was necessary.

Blood samples were aseptically collected using BACTEC culture bottles (Becton Dickinson, United States). For adult patients, both aerobic and anaerobic bottles were utilized, each containing 8–10 mL of blood, whereas pediatric samples were collected in aerobic bottles with 2–5 mL of blood. Immediately following collection, all specimens were inoculated into enriched BACTEC media and incubated in the BD BACTEC FX automated blood culture system for microbial growth detection, following the manufacturer′s standard protocol.

### 2.2. Culture and Isolation Procedures

When a culture bottle indicated a positive signal, an aliquot of the broth was subcultured onto blood agar and MacConkey agar plates and incubated aerobically at 37°C. The plates were examined daily, and those without visible growth after 96 h of incubation were considered negative, ensuring sufficient time for the recovery of both fastidious and slow‐growing organisms.

### 2.3. Identification and Preservation of Pathogens

Positive cultures were subjected to gram staining and biochemical characterization in accordance with standard microbiological procedures, whereas antimicrobial susceptibility testing was performed following the Clinical and Laboratory Standards Institute (CLSI, 2023) guidelines. Bacterial identification was primarily conducted using the BD Phoenix M50 automated system (Becton Dickinson, United States), which integrates biochemical profiling with antimicrobial susceptibility testing. Fungal isolates were identified using the DL‐96FUNGUS assay. For isolates that could not be conclusively identified through automated methods, conventional microbiological techniques were employed, including gram staining and a series of biochemical assays IMViC, catalase, coagulase, oxidase, urease, citrate utilization, and triple‐sugar‐iron (TSI) tests performed in accordance with the established laboratory manuals.

Following identification, isolates were preserved for subsequent antimicrobial susceptibility testing by transferring each confirmed isolate into tryptic soy broth supplemented with 20% glycerol and storing at –20°C to maintain viability for future confirmatory analyses.

### 2.4. Antibiotic Resistance Testing

Susceptibility testing was performed using both automated and manual methods to ensure precision and reliability. The BD Phoenix M50 system (Becton Dickinson, United States) was used for bacterial identification and susceptibility testing, whereas fungal isolates were analyzed using the DL‐96FUNGUS assay according to the manufacturer′s protocol. Susceptibility patterns were further verified using the Kirby–Bauer disk diffusion method on Mueller–Hinton agar, and results were interpreted following CLSI (2023) guidelines.

For the disk diffusion method, antibiotic disks (OXOID Ltd., United Kingdom) were used at standardized potencies. The antimicrobial agents tested included *β*‐lactams, comprising ampicillin (10 *μ*g) and amoxiclav (30 *μ*g), and cephalosporins representing successive generations: first‐generation cephradine (30 *μ*g), second‐generation cefuroxime (30 *μ*g), third‐generation cefixime (5 *μ*g), ceftazidime (30 *μ*g), ceftriaxone (30 *μ*g), cefotaxime (30 *μ*g), and fourth‐generation cefepime (30 *μ*g). Additional antibiotic classes evaluated were the aminoglycosides amikacin (30 *μ*g) and gentamicin (10 *μ*g), the macrolide azithromycin (15 *μ*g), the fluoroquinolone ciprofloxacin (5 *μ*g), and the glycylcyclines tigecycline (15 *μ*g). Antibiotic selection was based on their clinical relevance in treating BSIs, frequent empirical use in local healthcare settings, and the availability of CLSI interpretive breakpoints for standardized assessment.

All antimicrobial susceptibility testing procedures were conducted in accordance with CLSI quality‐assurance standards. To ensure analytical accuracy, reference quality‐control strains *E. coli* ATCC 25922 and *S. aureus* ATCC 25923 were included in each batch to validate media performance, disk potency, and inhibition zone measurements. Inhibition zones were recorded in millimeters and interpreted as susceptible, intermediate, or resistant based on CLSI (2023) interpretive criteria.

### 2.5. Data Analysis

Data on bacterial isolates, antimicrobial susceptibility patterns, and patient demographics were compiled and analyzed using Microsoft Excel (v16.0), whereas graphical representations were generated in R software (v4.5.0). Age was categorized into 12‐year intervals (0–12, 13–24, 25–36, 37–48, and > 49 years) to reflect developmental and physiological stages. Sex‐based variations were described descriptively, and since no inferential statistical tests were applied, these differences were interpreted as observational findings only.

### 2.6. Ethical Considerations

Ethical clearance was granted by the Institutional Ethical Committee of Vital Research Unit‐1. Informed consent was obtained from all participants or their guardians. All data were anonymized prior to analysis.

## 3. Result

### 3.1. Prevalence and Bacteriological Profile of BSIs

Out of a total of 498 blood specimens processed during the study period, 49 samples (9.84%) yielded positive bacterial growth, indicating bacteriologically confirmed cases of BSI. The remaining 449 samples (90.16%) showed no evidence of microbial growth following incubation and were classified as BSI negative.

Among the BSI positive cases, 25 isolates (51.0%) were recovered from male patients, whereas 24 isolates (49.0%) were obtained from female patients, indicating no substantial sex‐based disparity in BSI occurrence.

Age‐stratified analysis revealed that the highest proportion of isolates (36.7%) were recovered from patients aged above 49 years, followed by 18.37% from the 13–24 years age group, and 16.32% each from the 25–36 and 37–48 years cohorts. The lowest prevalence (12.25%) was observed in children aged 0–12 years (Table 1).

**Table 1:** Gender and age‐wise distribution of infected patients.

**Age**	**Gender**		**Total Patients (%)**
	**Male**	**Female**	
	**Patients (%)**	**Patients (%)**	
0–12	3 (6.1%)	3 (6.1%)	6 (12.25%)
13–24	5 (10.2%)	4 (8.2%)	9 (18.37%)
25–36	5 (10.2%)	3 (6.1%)	8 (16.32%)
37–48	5 (10.2%)	3 (6.1%)	8 (16.32%)
> 49	7 (14.3%)	11 (22.4%)	18 (36.7%)
Total	25 (51%)	24 (49%)	49

The 49 bacterial isolates were distributed across eight clinically significant taxa, encompassing both gram‐negative and gram‐positive bacteria. A total of 36 isolates (73.5%) were identified as gram‐negative bacilli, and 13 isolates (26.5%) as gram‐positive cocci. Among the gram‐negative isolates, the most frequently identified pathogen was *Salmonella* spp., accounting for 28.6% (*n* = 14) of all positive cultures, followed by *E. coli* (20.4%, *n* = 10), *Salmonella Typhi* (14.3%, *n* = 7), and *Klebsiella* spp. (10.2%, *n* = 5). The gram‐positive group was led by *S. aureus* (10.2%, *n* = 5), followed by *Streptococcus pyogenes* (6.1%, *n* = 3), *Streptococcus pneumoniae* (6.1%, *n* = 3), and *Staphylococcus epidermidis* (4.1%, *n* = 2) (Table 2).

**Table 2:** Prevalence of pathogens isolated on blood culture (*n* = 49).

**Gram stain group**	**Bacteria**	**Number**	**Percentage**
Gram‐negative	*Salmonella spp.*	14	28.6
	*E. coli*	10	20.4
	*Salmonella typhi*	7	14.3
	*Klebsiella spp.*	5	10.2

Gram‐positive	*Staphylococcus aureus*	5	10.2
	*Staphylococcus pyrogens*	3	6.1
	*Streptococcus pneumoniae*	3	6.1
	*Staphylococcus epidermidis*	2	4.1

### 3.2. AMR/Susceptibility Patterns of Blood Culture Isolates

Antibiotic susceptibility testing of 49 clinical bacterial isolates revealed heterogeneous resistance patterns across gram‐positive and gram‐negative species.

Gram‐negative bacteria demonstrated high levels of resistance to *β*‐lactam antibiotics. *E. coli* isolates (*n* = 10) exhibited the greatest resistance to cefuroxime (70%), amoxyclav (60%), and ampicillin (60%), while retaining higher susceptibility to amikacin and cephalosporins such as ceftazidime and ceftriaxone (70% each). *Klebsiella* spp. (*n* = 5) showed complete resistance to cefuroxime and limited susceptibility to tigecycline and gentamicin, indicating reduced treatment options. *Salmonella spp.* (*n* = 14) and *S. typhi* (*n* = 7) exhibited moderate‐to‐high resistance to multiple cephalosporins, ciprofloxacin, and ampicillin, with amikacin demonstrating the highest sensitivity (71.4%) (Figure 1).

Figure 1Antibiotic resistance pattern of gram‐negative bacteria (a) *E. coli,* (b) *Klebsiella* spp., (c) *Salmonella* spp., and (d) *Salmonella typhi*.

**Figure:**
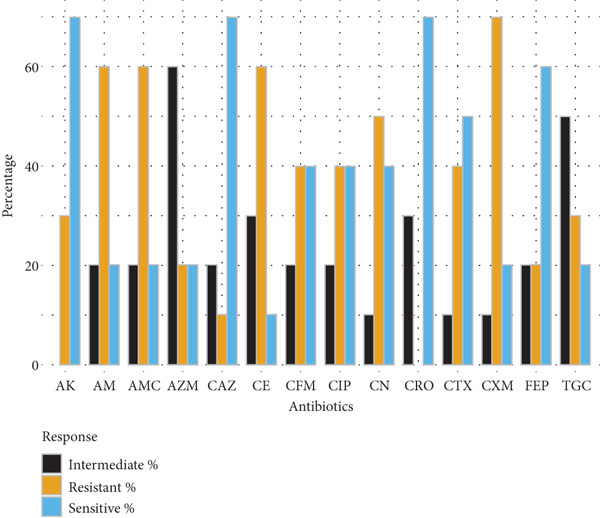
(a)

**Figure:**
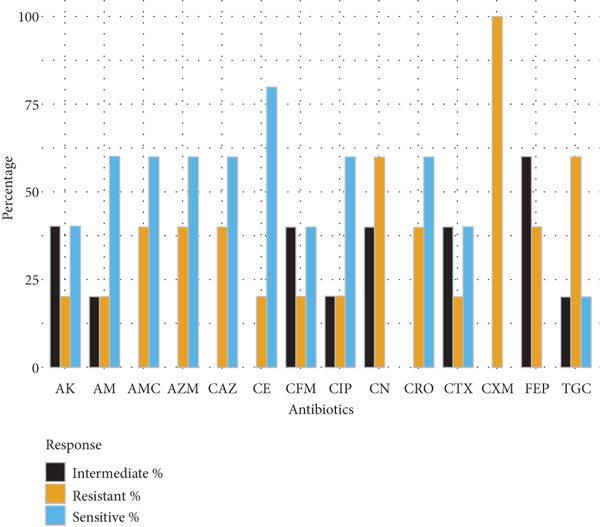
(b)

**Figure:**
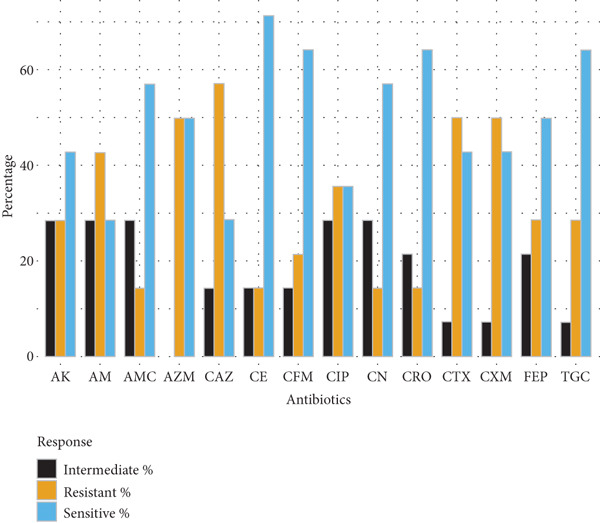
(c)

**Figure:**
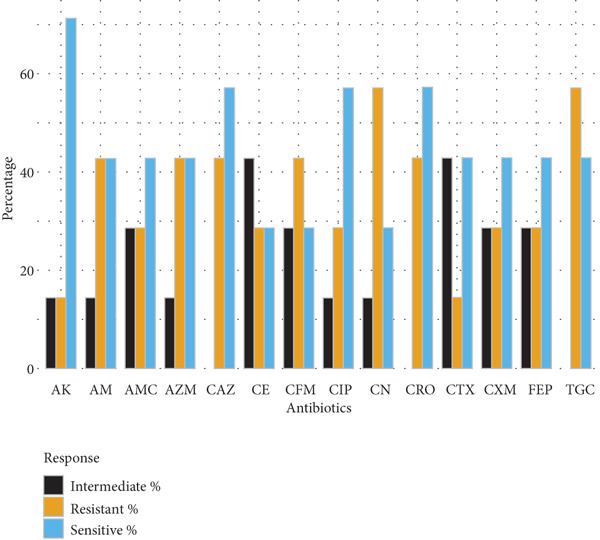
(d)

Gram‐positive cocci exhibited widespread resistance to third‐generation cephalosporins. *S. aureus* isolates (*n* = 5) were most resistant to cefotaxime and cefepime (80% each), with partial susceptibility retained for ceftazidime, ceftriaxone, and gentamicin (60%). *S. epidermidis* (*n* = 2) and *S. pneumoniae* (*n* = 3) displayed variable resistance patterns, with amikacin and ciprofloxacin remaining effective in some cases. *S. pyogenes* (*n* = 3) showed full susceptibility to cephradine but complete resistance to cefuroxime and cefotaxime (Figure 2).

Figure 2Antibiotic resistance pattern of gram‐positive bacteria (a) *Staphylococcus aureus,* (b) *Staphylococcus epidermidis,* (c) *Streptococcus pneumoniae, and* (d) *Streptococcus pyogenes.*

**Figure:**
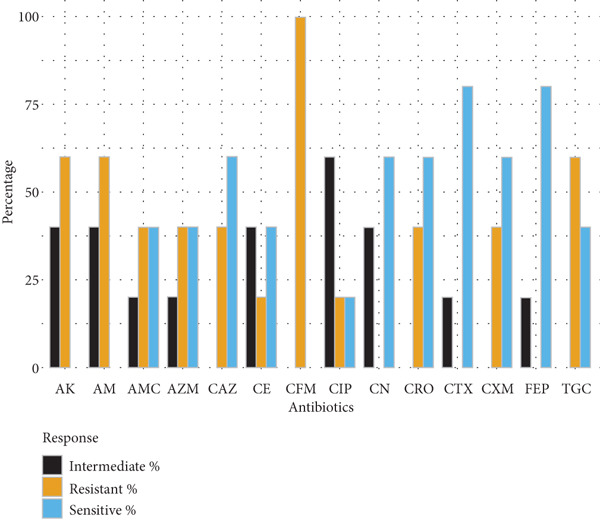
(a)

**Figure:**
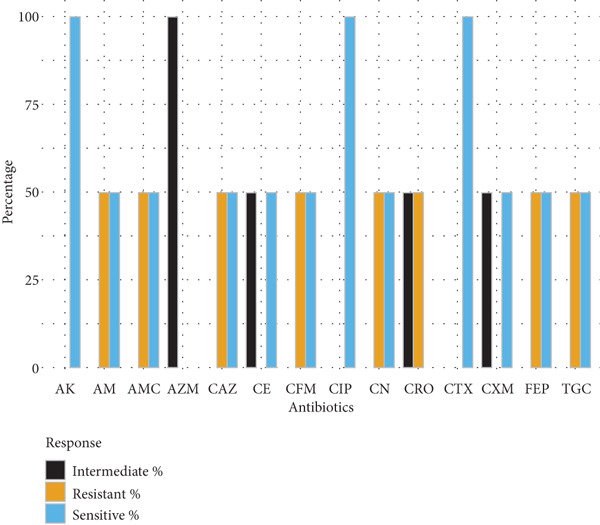
(b)

**Figure:**
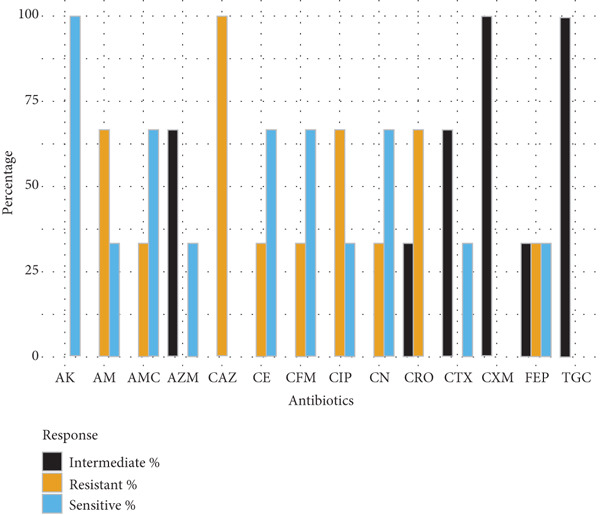
(c)

**Figure:**
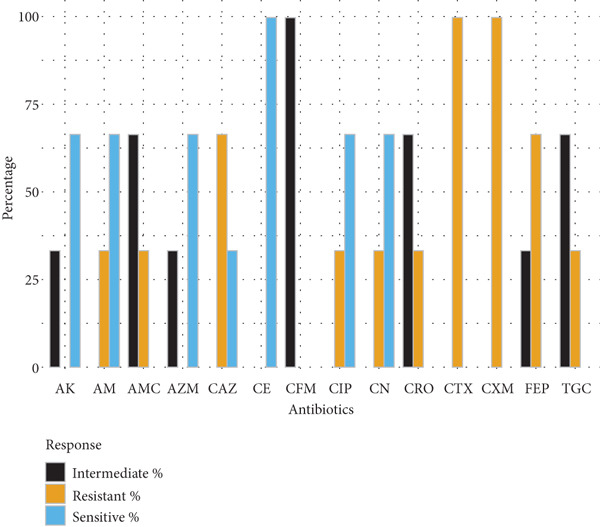
(d)

Overall, amikacin emerged as the most consistently effective agent across all bacterial species, whereas cefuroxime, ampicillin, and several extended spectrum cephalosporins exhibited high resistance rates.

## 4. Discussion

This study offers a timely exploration of BSIs and AMR dynamics in a semiurban region of Bangladesh, where community health practices, hospital transmission, and environmental exposures converge to shape infection outcomes. By aligning epidemiological patterns with microbiological evidence, our findings highlight both the local burden and the global interconnectedness of resistance evolution.

### 4.1. Epidemiology and Infection Burden

The 9.8% blood culture positivity observed here aligns with a systematic review of South and Southeast Asia, which reported overall rates near 9%, including 12% in adults and 7% in children [17]. This moderate yield likely reflects both the true burden of BSIs and common preanalytical limitations, such as prior antibiotic use and low‐blood volumes in resource‐limited laboratories [18]. Notably, the predominance of infection among individuals aged over 49 years (36.7%) points to the combined influence of immunosenescence, chronic comorbidities, and increased exposure to invasive procedures. Together, these factors heighten the risk of bloodstream infection in older adults, emphasizing the need for age‐specific surveillance and targeted preventive measures [19–21]. Moreover, comparable age‐related trends have been consistently documented in South Asian cohorts, underscoring the regional consistency of this pattern [22–26].

The dominance of gram‐negative bacteria (73.5%), particularly *Salmonella* spp.*, E. coli*, and *Klebsiella spp*., reflects a distinct epidemiological pattern compared with high‐income settings, where gram‐positive bacteria often predominate [27–29]. This distribution underscores the enteric and environmental origin of many BSIs in South Asia, where unsafe water, poor sanitation, and foodborne transmission remain endemic [30]. The high proportion of *Salmonella* spp. (28.6%) reinforces this interpretation and aligns with data from South and Southeast Asia linking BSIs to contaminated food and inadequate hygiene infrastructure [31].

The coisolation of *Klebsiella spp.* and *S. aureus* suggests possible nosocomial transmission, consistent with regional reports describing hospital‐associated BSIs caused by both gram‐negative and gram‐positive bacteria [32]. Thus, the findings reflect a dual epidemiological burden where enteric infections and hospital‐acquired pathogens coexist, driven by overlapping environmental and clinical pathways.

### 4.2. Resistance Landscape and Selective Pressures

The resistance landscape revealed in this study captures the accelerating collapse of conventional antibiotic efficacy within community and hospital ecosystems. The predominance of *β*‐lactam and cephalosporin resistance among *E. coli* and *Klebsiella* spp. mirrors a regional crisis where first‐line agents have lost their therapeutic reliability [32]. Such patterns are not confined to tertiary centers but increasingly emerge in semiurban populations, suggesting that the selective pressure driving AMR is deeply embedded in daily clinical and societal practices. In Bangladesh and across South Asia, antibiotics remain widely accessible without prescription, fostering chronic low‐level exposure that selects for resistant strains [33, 34]. The near‐universal reliance on empirical therapy often initiated before diagnostic confirmation amplifies this effect [35, 36]. Consequently, resistance becomes self‐reinforcing: Each treatment failure prompts escalation to higher‐generation antibiotics, compressing the therapeutic hierarchy and hastening the depletion of available options.

These findings illustrate how resistance is less a sudden phenomenon than a gradual ecological shift driven by behavioral, clinical, and structural determinants. The bloodstream, in this context, functions as a mirror of antimicrobial use in the community biological record of selective pressure diffused through human and environmental networks.

### 4.3. Ecological and Behavioral Drivers of Resistance

The resistance landscape in Feni reflects the cumulative effect of antibiotic misuse, weak regulation, and environmental dissemination. Over the counter antibiotic sales and incomplete dosing are common in Bangladesh, sustaining sublethal selective pressures that foster resistance [37, 38]. Concurrently, antibiotic residues and resistant bacteria in wastewater, aquaculture, and livestock effluents create environmental reservoirs that perpetuate horizontal gene transfer via integrons and plasmids [39].

This evidence supports the One Health framework, which posits that human, animal, and environmental health systems form an interconnected resistome [40]. The similarity between resistance profiles in community and clinical isolates from this study underscores this linkage: Resistant pathogens are no longer confined to hospitals but circulate within broader ecological networks. Addressing AMR in such contexts thus requires interventions that extend beyond hospitals to include agricultural policy reform, environmental sanitation, and antibiotic waste management.

### 4.4. Therapeutic Implications and Stewardship Priorities

Remarkably, amikacin remained the only antibiotic showing meaningful activity (> 70%) against multidrug‐resistant pathogens. This preserved efficacy may reflect restricted community exposure and reduced evolutionary pressure for aminoglycoside‐modifying enzymes. The result highlights a potential therapeutic window where judicious amikacin use could still yield clinical benefit. However, its nephrotoxicity and parenteral formulation confine its use to controlled clinical settings, reinforcing the need for careful stewardship. The widespread failure of *β*‐lactams, macrolides, and fluoroquinolones underscores the urgency of adaptive, evidence‐based prescribing guided by local antibiograms. Embedding diagnostic stewardship into clinical practice could ensure that empirical choices rapidly transition to targeted, data‐driven therapy, slowing the resistance trajectory.

### 4.5. Integrative Implications and Future Directions

The patterns uncovered in this study mirror a global transformation in resistance ecology. The convergence of community‐acquired and nosocomial pathogens in Feni exemplifies how AMR transcends boundaries driven by the same forces of microbial adaptation and human behavior described globally [41]. To mitigate this, Bangladesh must expand regional surveillance networks integrating clinical, veterinary, and environmental data, following WHO′s GLASS model. Policymakers should enforce prescription‐only antibiotic sales and incentivize laboratories to maintain standardized antibiogram reporting. Furthermore, future studies should incorporate whole‐genome sequencing to identify resistance genes and track clonal dissemination across the ecosystems. This molecular approach would bridge the gap between phenotypic resistance and genotypic understanding, enhancing prediction and prevention.

Overall, this study reveals that BSIs in Feni, Bangladesh, are not isolated clinical phenomena but reflections of a broader ecological crisis. The interplay between environmental contamination, empirical antibiotic use, and microbial gene exchange forms a self‐sustaining loop of resistance. These findings confirm that AMR is fundamentally a human and environmental challenge born of everyday practices, shaped by inequity, and sustained by neglect [42]. Breaking this cycle requires reimagining antibiotic use not as a solitary act of treatment but as a shared societal responsibility within a unified One Health framework.

## 5. Conclusion

This study illuminates how BSIs in semiurban Bangladesh capture the global dynamics of AMR: environmental exposure, hospital transmission, and antibiotic misuse coalescing into a single continuum. The predominance of drug‐resistant gram‐negative bacteria, especially *Salmonella* spp., *E. coli*, and *Klebsiella* spp., signals the erosion of conventional treatment efficacy and the encroachment of community resistance into hospital settings. Beyond the clinic, this work restates that AMR is not only a microbiological problem but a human and environmental crisis. It demands collective accountability through stronger policies, community education, and One Health integration. To preserve the effectiveness of existing antibiotics, Bangladesh and similar regions must transition from reactive treatment to proactive stewardship, turning surveillance into prevention and science into sustained, inclusive action.

## Disclosure

All authors read and approved the final manuscript.

## Conflicts of Interest

The authors declare no conflicts of interest.

## Author Contributions

All authors contributed to the study conception. Material preparation, data collection, and analysis were performed by Samim Mia and Md Zahirul Islam. The first draft of the manuscript was written by Samim Mia, Md Zahirul Islam, and Dr. Mohammad Zakerin Abedin, and other authors commented and modified the manuscript.

## Funding

No funding was received for this manuscript.

## Data Availability

The data that support the findings of this study are available on request from the corresponding author. The data are not publicly available due to privacy or ethical restrictions.
